# Ehrlichia-Induced Hemophagocytic Lymphohistiocytosis (HLH) With No Response to Doxycycline Treatment

**DOI:** 10.7759/cureus.42325

**Published:** 2023-07-23

**Authors:** Hana Qasim, Ammar Al-Obaidi, Abat Khan, Carl Freter

**Affiliations:** 1 Internal Medicine, University of Missouri–Kansas City, Kansas City, USA; 2 Hematology/Oncology, University of Missouri–Kansas City, Kansas City, USA; 3 Oncology, Saint Luke's Hospital of Kansas City, Saint Luke’s Cancer Institute, Kansas City, USA

**Keywords:** cytokine storming, secondary hemophagocytic lymphohistiocytosis (hlh), failure of oral doxycycline treatment, hemophagocytic lymphohistiocytosis (hlh), ehrlichia chaffeensis

## Abstract

Hemophagocytic lymphohistiocytosis (HLH) is a rare, life-threatening condition characterized by the persistent activation of antigen-presenting cells and multisystemic inflammation. Ehrlichiosis is a tick-born infection that primarily infects the white blood cells and can present with a variety of symptoms, including fever, fatigue, and multisystemic complications. Here, we present a 58-year-old female hospitalized for a urinary tract infection. Her hospital course was complicated by HLH, which was later discovered to be precipitated by an *Ehrlichia chaffeensis* infection. The patient did not respond to the doxycycline treatment, developed multiorgan failure, and passed away after a few weeks of treatment trials.

## Introduction

Hemophagocytic lymphohistiocytosis (HLH) is a hyperinflammatory, multisystemic disease with a widespread activation of antigen-presenting cells and CD8+ cells [1]. Infections are common triggers for both familial and sporadic cases, with viruses being the most commonly identified infections. In a previous case series of *Ehrlichia*-induced HLH, all patients recovered with no evidence of relapse after adequate treatment of the precipitating infection [2], which unfortunately was not the case in our patient. This might suggest using proapoptotic chemotherapy and immunosuppressive drugs even in infection-related HLH.

## Case presentation

We report a 58-year-old female patient with a past medical history of hypertension, treated hepatitis C, obesity, and recurrent urinary tract infection (UTI), who presented to the hospital complaining of malaise, anorexia, decreased urinary output, lightheadedness, and fever. She was initially diagnosed with UTI, was prescribed trimethoprim-sulfamethoxazole, and discharged home. She returned to the emergency department later that day with a worsening fever and was admitted to the hospital and started on IV antibiotics. The patient continued to have fever with hypotension and was later admitted to the critical care unit with septic shock, pneumonia, and hypoxemic respiratory failure. She was placed on a high-flow nasal cannula and was started on norepinephrine for worsening hypotension. Her respiratory status continued to decline, requiring bilevel positive airway pressure (BiPAP). Two days later, the patient developed generalized seizures with decreased responsiveness and was ultimately intubated for acute respiratory failure. She also developed acute kidney injury with tubular necrosis requiring emergent dialysis. The patient continued to be hypotensive, requiring norepinephrine and vasopressin.

**Figure 1:**
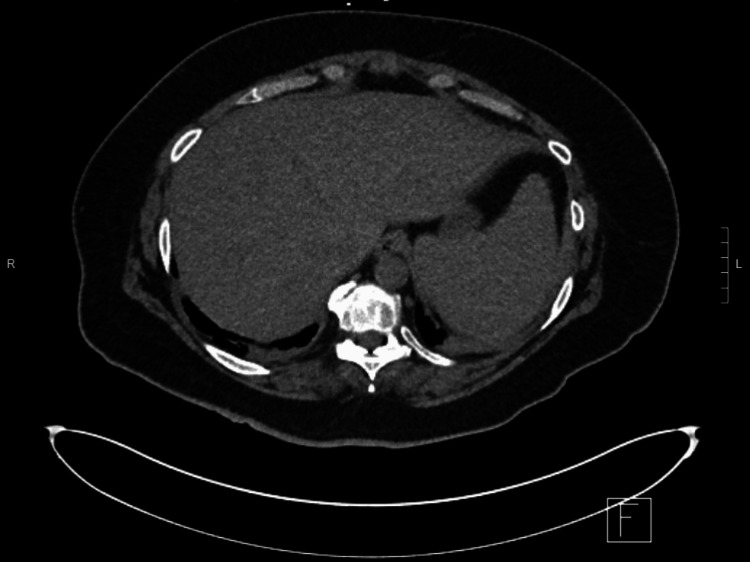
Hepatosplenomegaly with the liver measuring 23 cm craniocaudally and the spleen measuring 14.6 cm.

The hemato-oncology team was consulted due to concern for HLH syndrome, as the patient developed anemia, thrombocytopenia, hypofibrinogenemia, and deranged liver enzymes (Table 1). Two peripheral smears were done and did not demonstrate a microangiopathic hemolytic anemia picture. CT scan (Figure 1) showed hepatosplenomegaly with the liver measuring 23 cm craniocaudally and the spleen measuring 14.6 cm. The H score for HLH (HScore) is a scale to estimate the risk of having reactive hemophagocytic syndrome based on the patient's risk factors, symptoms, and lab tests. Patients with a score higher than 241 have more than a 99% probability of having HLH. Our patient's score was 269. Treatment for HLH was started with etoposide 112.5 mg/m^2^ (dose was reduced for acute kidney injury), along with dexamethasone 10 mg/m^2^ daily for two weeks and one dose of intravenous immunoglobulin (IVIG) and meropenem. Workup, including blood and urine cultures, ultimately revealed positive *Ehrlichia chaffeensis* PCR in the blood and *Enterococcus *UTI. The patient was started on doxycycline; her parameters started to improve with down-trending triglyceride and ferritin. With the confirmation of *Ehrlichia *infection, IVIG and etoposide were held, and the patient remained on high-dose dexamethasone for two weeks and then a tapering regimen. The patient required multiple transfusions of packed red blood cells, fresh frozen plasma, cryoprecipitate and platelets, and filgrastim injections throughout her hospital stay.

**Table 1:** Blood test results of the patient compared to normal values. AST: aspartate aminotransferase; INR: international normalized ratio

Blood test	Patient’s value	Normal value
Hemoglobin	6 g/dL	12- 15.1 g/dL
Platelet	9 Th/uL	150-450 Th/uL
White blood cells	0.8 TH/uL	4.5-11 TH/uL
AST	1,074 U/L	8-33 U/L
INR	1.5	0.8-1.2
Triglyceride	744 mg/dl	<150 mg/dl
Fibrinogen	83 mg/dl	200-400 mg/dL
Ferritin	>16,500.0 ng/mL	7.3-270 ng/mL
Interleukin-2 receptor	9798 U/ml	223-710 U/ml

The patient developed atrial fibrillation with a rapid ventricular response, treated with amiodarone. Her vasopressor requirements improved, and amiodarone, epinephrine, and vasopressin were weaned off, although she continued to have severe thrombocytopenia and anemia requiring transfusions. Fungal elements were present on the sputum culture and positive Fungitell >500; she was started on micafungin (Mycamine). She was extubated for two days and then re-intubated for respiratory distress. She had worsening hypotension and deranged liver enzymes with rising ferritin and bilirubin. High-dose dexamethasone was resumed for recurrent HLH. A magnetic resonance cholangiopancreatography (MRCP) was done for high bilirubin and deranged liver enzymes and showed possible pancreatitis with walled-off necrosis. The hemato-oncology team recommended a slow taper of steroids due to lab abnormalities likely caused by acute pancreatitis. The patient had worsening pneumonia and *Staphylococcus epidermidis* bacteremia. She continued to require vasopressors, and goals of care were discussed with the family. She was transitioned to a do-not-resuscitate (DNR) order and ultimately to comfort care and died.

## Discussion

HLH is a rare but life-threatening syndrome of excessive immune activation of normal but overactive histocytes and lymphocytes, which causes tissue destruction [3].

HLH can be either primary or secondary. Children are more likely to develop primary HLH, also known as familial/hereditary HLH, while adults are more likely to develop secondary HLH (sHLH), also known as sporadic/acquired HLH. While secondary HLH is generally linked to underlying infections, cancers, autoimmune illnesses, or drugs, primary HLH is mostly caused by genetic abnormalities [2,4].

The consistent etiology among all types of HLH is related to the defective function of natural killer (NK) cells and cytotoxic lymphocytes that normally eliminate the activated macrophages and CD+8 T cells via perforin-dependent cytotoxicity, where NK cells and cytotoxic T lymphocytes create pores in macrophage membranes and deliver protease-containing granules that can initiate the apoptosis process [1,3,5]. The unregulated activated macrophages secrete excessive amounts of cytokines and interferon-gamma, ultimately causing severe tissue damage and organ failure. Because of immune dysregulation, patients with immune deficiency disorders have an increased risk of HLH [5,6].

Multiple triggers have been identified for sHLH, with infections being the most common trigger in adults, followed by malignancy [2,6]. Viral infections are the most common triggers in the infectious group, predominantly Epstein-Barr virus (EBV) and human immunodeficiency virus (HIV) [2,6]. Less commonly, HLH is identified in bacterial and fungal infections [7].

Drug-induced HLH is another known but rare entity. Drugs that cause HLH are usually the ones that are known to cause hypersensitivity syndrome (drug rash with eosinophilia and systemic symptoms (DRESS)), such as lamotrigine or sulfamethoxazole, or immunotherapy medications, such as immune checkpoint inhibitors. 

Patients with HLH can present with non-specific signs and symptoms, which include fever, splenomegaly, rash, arthralgia, lymphadenopathy, and central nervous system (CNS) manifestations. They may also have a number of aberrant test results, such as cytopenia, hyperferritinemia, hypertriglyceridemia, and hypofibrinemia. Although no single clinical or laboratory criteria possesses the sensitivity and specificity necessary for an accurate diagnosis of HLH [4,6], a high level of ferritin should always trigger the inclusion of HLH in the differential diagnosis [6]. Ferritin levels >10,000 ug/L (microgram/L) are >90% sensitive and specific for HLH diagnosis in children but less so in adults [6].

HLH in adults should be diagnosed using the HLH-2004 diagnostic criteria [2,6] (Table 2). Another useful diagnostic tool is the HLH-probability calculator (HScore), an online calculator accessible over the web [6]. An underlying disease typically accompanies HLH in adults. A thorough investigation is required to rule out any underlying conditions, such as bacterial and viral infections, malignancies, and autoimmune diseases. The association between HLH and infections is significant because HLH has the potential to mimic infections and can make it difficult to diagnose infections that are curable when present [2,6].

**Table 2:** HLH-2004 diagnostic criteria IL-2 receptor: interleukin-2 receptor; HLH: hemophagocytic lymphohistiocytosis; NK: natural killer

The diagnosis of HLH can be established if Criterion 1 or 2 is fulfilled:
1. A molecular diagnosis consistent with HLH
2. Diagnostic criteria for HLH fulfilled (five of the eight criteria below)
Fever
Splenomegaly
Cytopenias, affecting >2 of the 3 lineages in the peripheral blood:
Hemoglobin <90 g/L
Platelets <100 x 10^9^/L
Neutrophils <1.0 x 10^9^/L
Hypertriglyceridemia and/or hypofibrinogenemia:
Fasting triglycerides >3.0 mmol/L (i.e., >265 mg/dl)
Fibrinogen <1.5 g/L
Hemophagocytosis in the bone marrow or spleen or lymph nodes. No evidence of malignancy.
Low or no NK cell activity (according to local laboratory reference)
Ferritin >500 ug/L
sCD25 (i.e., soluble IL-2 receptor) > 2400 U/ml

HLH has a high mortality rate of 58-75% in adults [2]. Treatments should be tailored to control hyperinflammation and treat the identified disease trigger, as early diagnosis and treatment have better outcomes [2,6].

*Ehrlichia *is obligate intracellular bacteria that grow within membrane-bound vacuoles in human and animal leukocytes. *E. chaffeensis*, which is one of the two most important species in humans, causes human monocytic ehrlichiosis (HME). HME is endemic in the south-eastern, mid-Atlantic, and south-central regions of the United States. HME is a tick-born disease, with the lone star tick being the most common vector. Transfusion-related cases are also reported.

The presentation of HME can vary, from subclinical to mild self-limited, to severe disease requiring hospitalization. Symptoms include fever, chills, malaise, headaches, myalgia, skin rash, altered mental status, and meningoencephalitis clinical picture, including severe headache, altered mental status, and seizures [8]. The skin rash in HME infection may range from maculopapular to petechial in nature, and it is usually not pruritic. Other forms of skin rash, such as blistering, nodular ,vasculitic, or ulcerated, have been described as well.

*Ehrlichia *and EBV, which are common triggers of HLH, infect the white blood cells, which may explain the hematological complications of both infections. *E. chaffeensis* is a rare trigger for secondary HLH, described in case reports, and one case series described a secondary HLH in adolescents and adults triggered by *E. chaffeensis* [2,9]. Early treatment with doxycycline in previously reported cases proved to be safe and effective in controlling the cytokine release storm and eradicating the infected cells, with full recovery and no evidence of relapse [2], which is, unfortunately, not the case in our patient. In another study for HME-related HLH in the pediatric population, among eight patients, three received chemotherapy and doxycycline, one of whom died; the other five were treated with doxycycline without chemotherapy, and all survived without HLH recurrence [10].

In another case report, a patient with a history of multiple myeloma, treated with autologous stem cell transplant and on maintenance therapy with daratumumab and pomalidomide and in stringent complete remission, died from HME-related HLH despite treatment with doxycycline [11]. In another case series, one patient got doxycycline without chemotherapy, while the other patient got doxycycline plus chemotherapy (etoposide and dexamethasone), and both recovered [12]. Similarly, in a case series of four patients including one child, two patients were treated with doxycycline, while the other two were treated with doxycycline and chemotherapy, and all recovered [13].

We believe that multiple factors have contributed to the unfortunate end results of our patient course, including sepsis, hospital-acquired pneumonia, and respiratory failure, which made it challenging at some points during her hospital course to differentiate between sepsis and HLH. Although it is more likely that HLH was induced by an *Ehrlichia* infection, other infection-induced HLH and drug-induced HLH remain less likely possibilities.

## Conclusions

Secondary HLH should be considered in patients with fever, worsening liver enzymes, and worsening cytopenia with no obvious cause. sHLH can be triggered by a variety of infections, including *E. chaffeensis*, which makes performing a comprehensive infectious work-up a significant step in the early diagnosis and treatment of this syndrome. In many cases of infection-induced HLH, patients can have more than one infection at the same time, which makes it challenging to identify which one is the trigger. Moreover, even with the use of broad-spectrum antibiotics to cover all the possible infections, treating the triggering infection is commonly not enough to break the cytokine release storm cycle. 

We propose that in infection-related HLH, if antibiotics are insufficient to stop the cytokine release storm early, then chemotherapy and immunosuppressive medications should be started with no delay, as antibiotics alone can be insufficient to stop the persistent macrophage and CD8+ T cell activation, and drugs targeting the hyperactivated T cells and histiocytes must be initiated.
